# Frailty and heart failure: State‐of‐the‐art review

**DOI:** 10.1002/jcsm.13306

**Published:** 2023-08-16

**Authors:** Khawaja M. Talha, Ambarish Pandey, Marat Fudim, Javed Butler, Stefan D. Anker, Muhammad Shahzeb Khan

**Affiliations:** ^1^ Department of Medicine University of Mississippi Medical Center Jackson MS USA; ^2^ Division of Cardiology University of Texas Southwestern Medical Center Dallas TX USA; ^3^ Division of Cardiology Duke University Hospital, Duke University School of Medicine Durham NC USA; ^4^ Duke Clinical Research Institute Durham NC USA; ^5^ Baylor Scott and White Research Institute Dallas TX USA; ^6^ Department of Cardiology (CVK) of German Heart Center Charité Institute of Health Center for Regenerative Therapies (BCRT), German Centre for Cardiovascular Research (DZHK) partner site Berlin, Charité Universitätsmedizin Berlin Germany; ^7^ Institute of Heart Diseases Wroclaw Medical University Wroclaw Poland

**Keywords:** cardiac rehabilitation, frailty, heart failure, medical therapy, prognosis

## Abstract

At least half of all patients with heart failure (HF) are affected by frailty, a syndrome that limits an individual ability to recover from acute stressors. While frailty affects up to 90% of patients with HF with preserved ejection fraction, it is also seen in ~30–60% of patients with HF with reduced ejection fraction, with ~26% higher prevalence in women compared with men. The relationship between frailty and HF is bidirectional, with both conditions exacerbating the other. Frailty is further complicated by a higher prevalence of sarcopenia (by ~20%) in HF patients compared with patients without HF, which negatively affects outcomes. Several frailty assessment methods have been employed historically including the Fried frailty phenotype and Rockwood Clinical Frailty Scale to classify HF patients based on the severity of frailty; however, a validated HF‐specific frailty assessment tool does not currently exist. Frailty in HF is associated with a poor prognosis with a 1.5‐fold to 2‐fold higher risk of all‐cause death and hospitalizations compared to non‐frail patients. Frailty is also highly prevalent in patients with worsening HF, affecting >50% of patients hospitalized for HF. Such patients with multiple readmissions for decompensated HF have markedly poor outcomes compared to younger, non‐frail cohorts, and it is hypothesized that it may be due to major physical and functional limitations that limit recovery from an acute episode of worsening HF, a care aspect that has not been addressed in HF guidelines. Frail patients are thought to confer less benefit from therapeutic interventions due to an increased risk of perceived harm, resulting in lower adherence to HF interventions, which may worsen outcomes. Multiple studies report that <40% of frail patients are on guideline‐directed medical therapy for HF, of which most are on suboptimal doses of these medications. There is a lack of evidence generated from randomized trials in this incredibly vulnerable population, and most current practice is governed by post hoc analyses of trials, observational registry‐based data and providers' clinical judgement. The current body of evidence suggests that the treatment effect of most guideline‐based interventions, including medications, cardiac rehabilitation and device therapy, is consistent across all age groups and frailty subgroups and, in some cases, may be amplified in the older, more frail population. In this review, we discuss the characteristics, assessment tools, impact on prognosis and impact on therapeutic interventions of frailty in patients with HF.

## Introduction

Frailty is defined as a decline in an individual's physical and cognitive reserve that prohibits regulatory bodily mechanisms to counter or recover from an acute stressor driven by the amplification of inflammatory mechanisms and sarcopenia among other causes. Frailty is comprised of a multidimensional domain of physical, cognitive, social and mental aspects and is considered a risk enhancer in heart failure (HF) affecting ~40–80% of all HF patients.^1^, ^2^, ^3^ Frailty is more prevalent in patients with HF with preserved ejection fraction (HFpEF) affecting up to 90% of patients^4^ because the HFpEF phenotype occurs more commonly in elderly patients, a population that is inherently predisposed to frailty due to longevity and accumulation of other contributing co‐morbidities.^4^ Nevertheless, frailty is prevalent in 30–60%^5^, ^6^ of patients with HF with reduced ejection fraction (HFrEF) and is being increasingly recognized as a distinct biological syndrome causing physical and cognitive limitations, irrespective of age^7^ and the presence of other co‐morbidities.^8^ Frailty in HF is associated with a poor prognosis with a 1.5‐fold to 2‐fold higher risk of all‐cause death and hospitalizations,^5^, ^9^ and there is evidence to suggest that frailty is potentially a more important prognostic indicator compared to other traditional cardiovascular risk factors.^10^ Herein, we discuss physical frailty in HF, the mechanisms for development of frailty and its impact on outcomes, frailty assessment tools and the effectiveness of treatment interventions in frail HF patients.

## Mechanisms for frailty in heart failure

**Figure 1:**
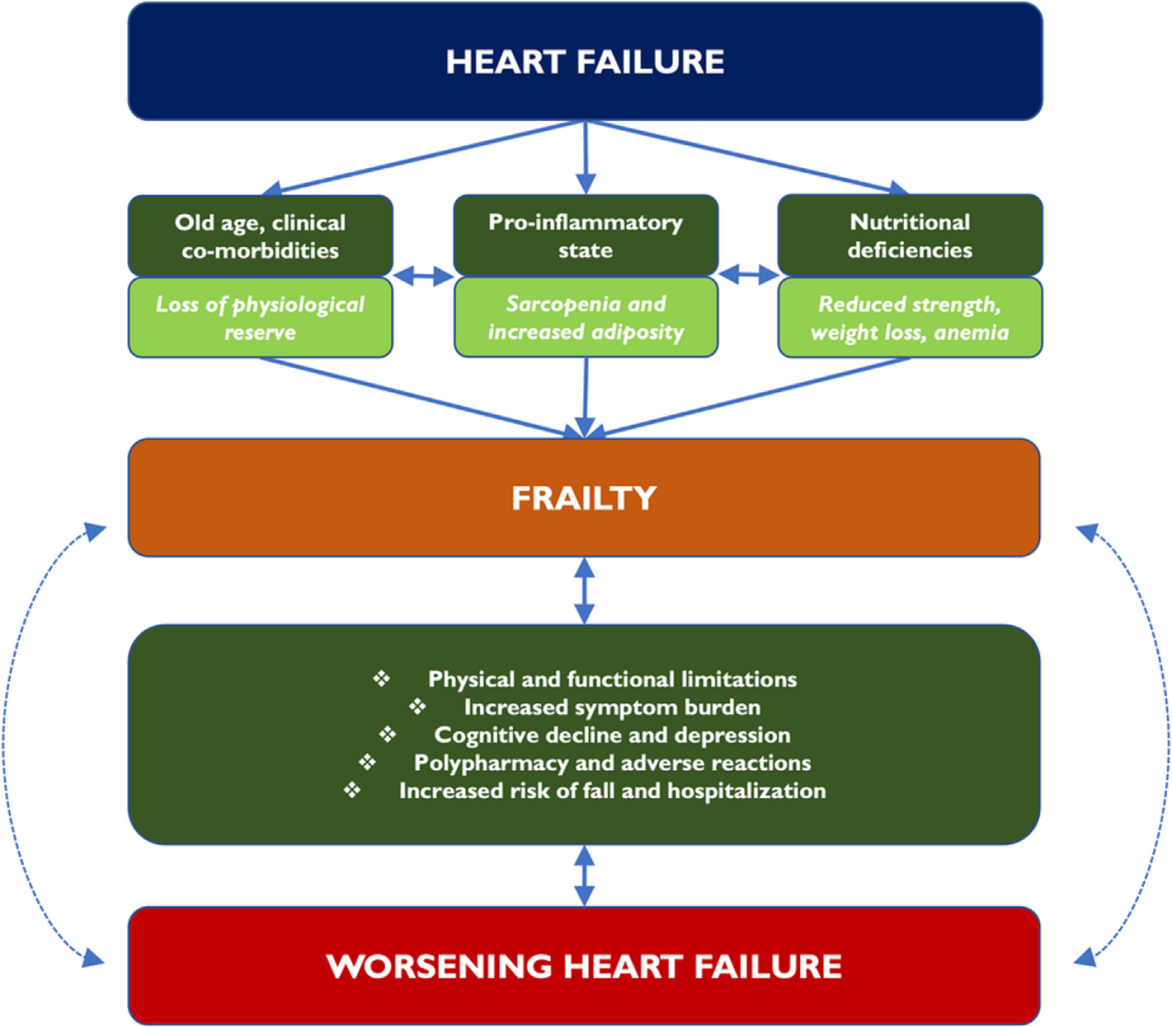
A pictorial illustration of the relationship between heart failure and frailty and how frailty in heart failure leads to worse outcomes.

HF is a major disease with multi‐system effects that impact the overall health of patients increasing their risk of frailty. HF is associated with a higher burden of co‐morbidities compared to the general population and also occurs largely in older individuals, so frailty may be a consequence or an indicator of poor health status. HF produces a pro‐inflammatory state due to neurohormonal and adrenergic overstimulation, which causes pathological changes in muscle composition, leading to loss of muscle mass and muscle strength and contributing to physical frailty.^11^ The heightened pro‐inflammatory state coupled with multi‐organ damage associated with congestion and hypoxia associated with HF, and the development of other co‐morbidities, for example, chronic kidney disease, result in loss of physiological reserve, which in turn contributes to frailty.^12^ Pro‐inflammatory markers, such as C‐reactive protein, tissue necrosis factor and interleukin‐6 (IL‐6), are elevated in HF and are associated with a heightened risk of HF in the elderly.^13^ Specifically, IL‐6 has been found to be more prevalent in elderly patients with a higher burden of cardiovascular co‐morbidities and is associated with increased mortality in HF.^14^ A post hoc analysis of the PARAGON‐HF (Prospective Comparison of ARNI [angiotensin receptor–neprilysin inhibitor] with ARB [angiotensin receptor blocker] Global Outcomes in HF with Preserved Ejection Fraction) trial showed an increasing neutrophil‐to‐lymphocyte ratio, a marker of systemic inflammation, with worsening frailty in HF patients.^15^ Systemic inflammation also leads to progression of atherosclerosis and insulin resistance, and changes in metabolic hormonal activity such as insulin growth factor and growth differentiation factor 15 (GDF‐15), which also contribute towards worsening frailty.^16^, ^17^ Higher levels of serum natriuretic peptides and high‐sensitivity troponin are also observed in frail patients reflecting worsening and ongoing myocardial injury leading to higher clinical adverse event rates. Nutritional intake is usually impaired in HF patients due to early satiety, malabsorption secondary to intestinal oedema, worsening symptoms of congestion, and depression or cognitive dysfunction in addition to HF‐related dietary limitations, contributing towards frailty.^18^ Low protein intake in HF patients has also been associated with increased congestion and higher mortality.^19^ Cognitive frailty, defined as co‐existence of cognitive impairment and physical impairment without neurogenerative dysfunction, is highly prevalent in patients with HF (approximately one in four elderly HF patients) and is associated with a >1.5‐fold increase in mortality and HF hospitalizations.^20^ Similarly, social frailty characterized as reduction in involvement in social activities is prevalent in two thirds of elderly patients with HF. Patients with social frailty are also more symptomatic at baseline and have higher rates of all‐cause mortality and HF hospitalizations.^21^ Psychological frailty is associated with incident HF, and common psychiatric illnesses like anxiety and depression are associated with a higher risk of HF hospitalization and death.^22^ The OPERA‐HF (Observational registry to assess and PrEdict the in‐patient course, risk of Re‐Admission and mortality for patients hospitalized for or with Heart Failure) study of 671 patients with mean age of 76 years found that cognitive and psychosocial decline were associated with recurrent HF hospitalizations and death following an HF hospitalization^23^ (*Figure* *1*).

## Frailty assessment in heart failure

Several methods have been employed in standardizing the definition of frailty in patients with HF, mostly for purposes of prognostication and studying differential therapeutic benefits of relevant interventions in clinical trials.^24^ A systematic review identified 67 instruments that have been employed to measure frailty, and the two most cited scales are the Fried frailty phenotype (also known as the patient frailty phenotype) and the deficit accumulation index proposed by Rockwood and colleagues.^25^

The Fried frailty phenotype was pioneered by Fried et al. and has been widely adopted in medical literature for frailty assessment.^26^ The initial work was based on using several markers of frailty that had been reported in previous literature using scientific rationale surrounding the ‘cycle of frailty’ and forming criteria using surrogate indicators to accurately ascertain an individual's frailty status. The five components included in the initial criteria were shrinking (unintentional weight loss, ≥10 lbs or ≥5% of body weight in the prior year), weakness (handgrip strength, lowest 20th centile), poor endurance and energy (self‐reported using an objective scale), slowness (lowest 20th centile, adjudged by time to walk 15 ft) and low physical activity (a weighted score of energy expended per week). The approach classified people as frail (≥3 deficits), pre‐frail (1–2 deficits) or non‐frail (no deficits). The original criterion has since undergone multiple iterations, with the modified Fried criterion used more commonly in recent times.^27^ It utilizes a population‐independent approach to the measurement of frailty using the aforementioned parameters to neutralize the effect of demographic variations to ascertain frailty more accurately. The Fried method has several limitations, including low generalizability of the scale to the real‐world population and lack of inclusion of cognitive assessment. However, the methods continue to be widely used in clinical trials and observational research.

Rockwood et al. proposed the Clinical Frailty Scale in 2005, which incorporated the two former methods of frailty grading and derived a 7‐point scale that ranged from 1 indicating robust health to 7 indicating complete functional dependence on others.^28^ Subsequently, the group proposed a deficit accumulation model to devise a frailty index (FI) that is currently used widely in frailty assessment and stratification in HF trials.^29^ This scale focuses on the incremental physical deficits that an individual acquires with age, with little regard to the nature or cause of those deficits. The authors argued that this approach is closest to the truest sense of frailty defined as a loss of physiological reserve regardless of co‐morbid conditions or reasons for physical limitations and accounts for cognitive deficits. The deficits need to fulfil a 5‐point domain to be eligible for inclusion and can represent symptoms, signs, disabilities and diseases. Moreover, a scale should contain at least 30–40 clinical deficits to be reliable. This method has been widely adopted due to a correlation with prognosis and used rather successfully in HF studies to stratify study populations based on the severity of frailty.^29^ Differences between the Fried frailty phenotype and Rockwood Clinical Frailty Scale are shown in *Table* *1*.

**Table 1:** A comparison of benefits of the Fried frailty phenotype and Rockwood Clinical Frailty Scale for frailty assessment

	Fried frailty phenotype	Rockwood Clinical Frailty Scale
Components	A fixed 5‐point assessment of signs and symptoms experienced by patients to assess physical frailty	Clinical evaluation of multiple patient co‐morbidities and physical and cognitive deficiencies in activities of daily living
Evaluation	Can be performed during routine clinical visit without a comprehensive clinical assessment	Needs thorough evaluation through a comprehensive multi‐domain clinical assessment
Classification	Categorizes phenotype into frail, pre‐frail and non‐frail categories based on a scoring system	Continuous variable covering the complete spectrum of ‘very fit’ to ‘severely frail’ phenotype
Advantages	Quick and easy to perform and interpret Utility in screening of frailty and pre‐frailty status	Assesses clinical, physical and cognitive domains of frailty Utility in temporal assessment of changes in frailty status over time Can be used in assessing frailty in patients that are functionally disabled
Limitations	Only assesses physical domain of frailty Inability to longitudinally track changes in frailty status Limited utility in patients that are functionally disabled	Reported as a continuous variable that can be difficult to interpret for clinicians Requires a comprehensive, holistic assessment of patient's clinical, physical and functional status

Other scales have been employed to specifically prognosticate the effect of frailty on outcomes in HF. Yamada et al. studied the accuracy of Frailty‐based prognostic criteria in HF patients (FLAGSHIP) scoring system in 2271 patients hospitalized for HF.^30^ Physical frailty was measured using a panel of four objective measurable components of physical activity—usual walking speed, grip strength, Performance Measure for Activities of Daily Living (PMADL‐8) and Self‐Efficacy for Walking‐7 (SEW‐7). Each measure was further stratified for points scoring depending on how well the individual performed, and a combined score was calculated, which was further classified into four categories. Compared to Category 1 (least frail), patients in Category 3 and Category 4 (most frail) of this novel frailty scale had significantly higher rates of the composite endpoint of all‐cause death and HF readmissions (Category 3: hazard ratio [HR] 2.37 [95% confidence interval—CI 1.32–4.23]; Category 4: HR 2.66 [95% CI 1.45–4.89]).

Although not currently part of existing frailty assessment tools, novel biomarkers could also be used to assess frailty and have a prognostic role in patients with HF. GDF‐15 is a stress‐responsive cytokine released in response to tissue injury and has been found to be a strong predictor of impaired mobility^31^, ^32^; hence, it is considered a core biomarker of frailty in older population.^33^, ^34^ GDF‐15 levels also are elevated in patients with cardiovascular disease, notably HF, and are associated with increased morbidity and mortality.^35^ MicroRNAs have also been found to be sensitive biomarkers of cognitive ageing, sarcopenia and frailty, and additionally, some of those plasma profile changes represent worsening cardiac fibrosis, hypertrophy and cardiac cell apoptosis in HF.^36^, ^37^ Procollagen type III N‐terminal peptide (P3NP) and synaptosomal‐associated protein of 25 kDa (SNAP25) are by‐products of collagen synthesis. Elevated levels of P3NP and SNAP25 are found in elderly patients and sarcopenia^38^ and are considered indicators of worsening physical performance in HF.^39^

Diagnostic codes‐based frailty assessment may be as reliable as objective frailty scales. Kohsaka et al. evaluated the use of International Classification of Diseases 9 (ICD‐9) diagnostic codes for assessment of frailty using the Department of Veteran Affairs database.^40^ Diagnostic codes that were queried included 781.2 (abnormality of gait), 783.2 (abnormal loss of weight/underweight), 783.7 (adult failure to thrive), 799.4 (cachexia), 799.3 (debility), 719.7 (difficulty in walking), V15.88 (fall), 780.7 (malaise and fatigue), 728.2 (muscular wasting and disuse atrophy), 728.87 (muscle weakness), 707.0, 707.2 (pressure ulcer) and 797 (senility without mention of psychosis). Patients with these diagnostic codes recorded had a higher 1‐year mortality rate (28.1% vs. 9.1% in non‐frail) and 1‐year all‐cause hospitalizations (79.5% vs. 58.1% in non‐frail), with findings consistent across the left ventricular ejection fraction (LVEF) spectrum.

Although several methods exist for frailty assessment as described above, none comprehensively encompass all important domains of frailty with a predominant focus on physical frailty measures. The Heart Failure Association of the European Society of Cardiology has proposed developing a multi‐domain frailty score for patients with HF comprising of clinical (presence of clinical co‐morbidities, weight loss and falls), physical–functional (ability to perform activities of daily living, mobility and balance), cognitive–psychological (cognitive impairment, dementia and depression) and social components (living alone, social support and institutionalization).^41^ This novel scoring system aims to provide utility in both clinical and research settings, be cheap and easy to perform in routine clinical care and be able to track longitudinal changes in frailty status in HF patients.

## Clinical characteristics and outcomes of heart failure patients with frailty

HF patients with frailty are a more vulnerable, high‐risk population, as they are more likely to be older and have a greater burden of co‐morbidities. Women with HF are more likely to be frail compared to men,^42^ which in part is attributed to lower body muscle mass and longer life expectancy. In a meta‐analysis of 29 studies and 8854 patients with HF, women were found to be at a 26% higher risk of being frail compared to men.^43^

A combined analysis of the PARADIGM‐HF (Prospective Comparison of ARNI with ACEi to Determine Impact on Global Mortality and Morbidity in Heart Failure) and ATMOSPHERE (Aliskiren Trial of Minimizing Outcomes in Patients With Heart Failure) trials was performed to assess the prevalence of frailty and associated baseline characteristics in 13 265 patients with HFrEF.^5^, ^44^, ^45^ Patients were classified into frail (FI > 0.210) and non‐frail groups (FI < 0.210) using a 42‐point deficit accumulation approach. Frailty was associated with increasing age, female sex and White race. Frail patients were more likely to have HF secondary to ischaemic aetiology, had a higher burden of cardiovascular co‐morbidities and had a higher number of implantable cardiac devices. They also had a higher New York Heart Association (NYHA) Class 3/4 percentage scores and lower median Kansas City Cardiomyopathy Questionnaire—Clinical Summary Score (KCCQ‐CSS). Frail patients had a higher risk of cardiovascular deaths, HF hospitalizations, all‐cause deaths and all‐cause hospitalizations. Study drug discontinuation was higher in the frail population compared to non‐frail patients. However, the drug's treatment effect in the PARADIGM‐HF trial was consistent across both frail and non‐frail groups.

The FRAGILE‐HF (Prevalence and Prognostic Value of Physical and Social Frailty in Geriatric Patients Hospitalized for HF: A Multicentre Prospective Cohort Study) was a prospective evaluation of baseline characteristics, the association of individual components of frailty assessment and outcomes in non‐dependent elderly patients with HF.^46^ The study enrolled 416 patients of whom 316 (76%) were frail using the Fried criteria, and ~50% of patients had LVEF < 50%. Frail patients had a higher prevalence of depression and worse health literacy scores and were prescribed with fewer HF medications than non‐frail patients, for example, angiotensin‐converting enzyme inhibitor (ACEi)/ARB and beta‐blockers. Frail patients had a >2‐fold risk of 1‐year mortality and a nearly 2‐fold increased risk of 1‐year all‐cause readmissions. A meta‐analysis by Yang et al. evaluated all‐cause mortality (eight studies) and hospitalizations (six studies) in frail patients compared to non‐frail patients with HF.^9^ Frailty was associated with a higher risk of all‐cause mortality (HR 1.54 [95% CI 1.34–1.75]) and all‐cause hospitalizations by a similar magnitude (HR 1.56 [95% CI 1.36–1.78]) over 1.8‐ and 1.1‐year follow‐up, respectively.

Patients with baseline cardiovascular co‐morbidities with high frailty are also at increased risk of incident HF. An analysis of the Look AHEAD (Action for Health in Diabetes) trial evaluated the incidence of HF in patients with diabetes stratified by level of frailty, which was measured using a 38‐point deficit accumulation index, with patients divided into low, intermediate and high frailty groups.^47^ When evaluated as a continuous measure, increasing frailty was associated with a high incidence of overall HF, with similar effects observed in both HFpEF and HFrEF. When evaluated as a categorical measure, high frailty was associated (not intermediate frailty) with a higher risk of incident HF, both HFpEF and HFrEF, when adjusted for age, sex, race/ethnicity and treatment arm.

## Frailty in patients with decompensated heart failure

Older and more frail patients with multiple readmissions for decompensated HF appear to have markedly poor outcomes compared to younger cohorts. It may be attributed to physical and functional limitations that limit recovery from an acute episode of decompensated HF, a care aspect that has not been addressed in HF guidelines. Reeves et al. prospectively evaluated frailty (assessed using the Fried frailty phenotype), physical and cognitive function, and quality of life (QOL) metrics in patients aged >60 years hospitalized for acute decompensated HF, compared to an age‐matched cohort of ambulatory patients with stable HF.^48^ Frailty was highly prevalent in the hospitalized cohort (56%) compared to no frailty in the group with age‐matched, stable, chronic HF. Hospitalized patients also had marked deficits in physical function in all domains, including Short Physical Performance Battery (SPPB) score and 6‐min walk testing. Cognitive dysfunction and depression were also significantly more prevalent among the hospitalized cohort and had higher KCCQ‐CSS scores. Another analysis from the REHAB‐HF (Rehabilitation Therapy in Older Acute Heart Failure Patients) trial revealed that of the first 202 patients with acute decompensated HF (age ≥60 years) enrolled in the study, 98% of patients were either frail or pre‐frail, as assessed by the Fried criteria.^49^ Compared to pre‐frail patients, frail patients had worse KCCQ overall summary scores (35 ± 19 vs. 46 ± 21) and had a higher prevalence of depression (53% vs. 40%). A secondary analysis of 1212 patients from the FRAGILE‐HF trial found that patients considered socially frail (defined on a 5‐point scale) were at a higher risk for 1‐year all‐cause mortality and HF hospitalizations.^21^

## Sarcopenia

While both sarcopenia and frailty share similar risk factors (e.g., malnutrition, hormonal changes and physical activity) and co‐exist with each other, they are considered distinct entities. Frailty is a multi‐system syndrome that represents a dynamic progression towards both physical and physiological decline, whereas sarcopenia is specifically defined as a loss of muscle mass and/or function and contributes towards physical frailty.^50^, ^51^ There is a higher prevalence of sarcopenia (by ~20%) in HF patients compared to non‐HF patients of the same age and is independently associated with worse clinical outcomes in HF.^52^, ^53^, ^54^ An observational study of 418 patients hospitalized for HF by Konishi et al. reported a significantly higher rate of mortality adjusted for age, sex, haemoglobin, NYHA class and height among patients with lower mean appendicular skeletal muscle mass and fat indices (0.825 [95% CI 0.747–0.908] per 1‐kg increase of skeletal mass and 0.954 [95% CI 0.916–0.993] per 1‐kg increase of fat mass).^55^ Similarly, a prospective cohort study of 268 patients, of which 17.5% had muscle wasting, evaluated the effect of low muscle mass on mortality in HF across the LVEF spectrum.^54^ Patients with muscle wasting were older, had higher N‐terminal pro‐b‐type‐natriuretic peptide (NT‐proBNP) levels and were more likely to be iron‐deficient. These patients also had a higher risk of death over a mean follow‐up of 67 months (HR 1.80 [95% CI 1.01–3.19]), with the effect more pronounced in HFrEF compared to HFpEF. In both these studies, skeletal muscle mass was evaluated using a dual‐energy X‐ray absorptiometry scan; however, several other methods have been used to evaluate muscle mass in patients with HF (e.g., abdominopelvic computed tomography scans and psoas muscle assessment) as markers of frailty. A post hoc analysis of 943 patients in the FRAGILE‐HF study evaluated the association of sarcopenia measured using handgrip strength, gait speed and appendicular skeletal mass, with mortality and HF readmissions in HF patients aged >65 years.^56^ The study found a similar prevalence of sarcopenia in both HFpEF and HFrEF and a significantly higher 1‐year mortality in patients with sarcopenia across the LVEF spectrum with no inter‐group differences between HFrEF and HFpEF.

## Cardiac rehabilitation as a parallel strategy to drug and device therapy

One of the major characteristics of frailty is marked physical and functional limitations. Physical limitation is highly prevalent in elderly HF patients—a post hoc analysis of the FRAGILE‐HF study in HF patients aged >65 years revealed that low physical performance, defined as low gait speed, delayed chair stand test and abnormalities in balance testing, was observed in ~84% of patients with HF.^57^ All individual and collective parameters were in turn associated with decreased exercise capacity measured by 6‐min walk testing. No significant differences were observed between patients with HFrEF and HFpEF. Another post hoc analysis of the REHAB‐HF trial assessed differences in physical performance, functional status and cognitive function between HFpEF and HFrEF.^58^ A total of 202 participants aged ≥60 were enrolled following HF hospitalization and were evaluated for frailty using the Fried criteria, cognitive function and health‐related QOL. The study reported that almost 50% of the combined cohort was frail, 75% had significant cognitive dysfunction and 50% had depressive symptoms. No significant difference in prevalence of frailty prevalence and cognitive dysfunction was found between HFrEF and HFpEF; however, patients with HFpEF were more likely to have depressive symptoms and worse health‐related QOL metrics.

Nevertheless, continued aerobic exercise and physical rehabilitation have been associated with improved clinical outcomes in frail HF patients. The original HF‐ACTION (Heart Failure: A Controlled Trial Investigating Outcomes of Exercise Training) trial evaluated the efficacy of aerobic exercise training in HFrEF in a relatively younger population (median age 59), and the results were only positive in favour of a reduction of in the primary composite of all‐cause mortality and hospitalizations when adjusted for highly prognostic variables (exercise duration, LVEF, depression and atrial fibrillation).^59^ The trial included ~700 patients over the age of 70 years, but the baseline characteristics did not align with those in real‐world elderly HF patients that also have a significant clinical co‐morbidity burden. The trial also did not assess strength training, balance testing and high‐intensity interval training, which are considered important parameters to assess in patients undergoing cardiac rehabilitation.^60^ Pandey et al. evaluated the effect of frailty on the efficacy of supervised aerobic exercise training as a post hoc analysis from the HF‐ACTION trial.^6^ A total of 2130 participants were divided into high and low frailty subgroups using the deficit accumulation index and followed for a median follow‐up of 2.9 years. The study found a significant reduction in the primary composite outcome with exercise training in frail patients (HR 0.83 [95% CI 0.72–0.95]) majorly driven by a reduction in all‐cause hospitalizations, whereas no significant reduction was observed in non‐frail patients (HR 1.04 [95% CI 0.87–1.25]). The reduction in the primary endpoint was majorly driven by a reduction in all‐cause hospitalizations. A significant improvement in QOL from baseline as measured by the KCCQ‐CSS score was observed; however, no between‐group differences were present between the frail and non‐frail groups.

The REHAB‐HF trial evaluated the efficacy of a structured physical rehabilitation programme in enhancing recovery in 349 elderly patients hospitalized for HF that were ambulatory and functionally independent at baseline.^61^ The intervention focused on strength, balance, mobility and endurance and was started in‐hospital and continued post‐discharge. Mean age of patients was 73 ± 8 years, and 97% were frail based on the modified Fried criteria. There was a significant improvement in the SPPB score at 3 months in the intervention arm (mean between‐group difference, 1.5; 95% CI 0.9–2.0; *P* < 0.001), which persisted after adjusting for the diabetes and peripheral vascular disease. There were also significant improvements observed in 6‐min walk testing, frailty status, KCCQ‐CSS and depression in the intervention group; however, no differences in all‐cause mortality, all hospitalizations and HF hospitalizations were seen between both groups. In a post hoc analysis, Mentz et al. found that the effect was consistent across patients with HFrEF (LVEF < 45%) and HFpEF (LVEF ≥ 45%).^62^ A multi‐centre retrospective study in Japan evaluated the effects of cardiac rehabilitation in 3277 ambulatory HF patients and found a significant reduction in all‐cause mortality and HF admissions in patients that underwent outpatient cardiac rehabilitation compared with those who did not, with a consistent effect observed in patients with HFpEF or frailty.^63^

These data suggest that cardiac rehabilitation plays a crucial role in improving functional patient‐reported outcomes in frail patients with HF. The 2022 American Heart Association/American College of Cardiology/Heart Failure Society of America guidelines denote a Class 1 indication for exercise training and Class 2a indication for a cardiac rehabilitation programme for improving functional status, exercise tolerance and health‐related QOL.^64^ However, utilization of cardiac rehabilitation is low. A recent analysis of the Get With the Guidelines—Heart Failure registry reported that only one tenth of all patients hospitalized for HF are referred for cardiac rehabilitation at discharge with a modest rising trend observed in recent years.^65^ Potential reasons for this include high financial costs of enrolling in the programme, transportation‐related issues and lack of awareness among participants about benefits of cardiac rehabilitation.^66^ Initiatives like systematic referral, dissemination of information regarding the structure and benefits of the programme through pamphlets and discussions at bedside, and incorporation of automated order sets coupled with patient discussion at discharge have shown promise in improving enrolment.^67^, ^68^, ^69^ Moreover, home‐based cardiac rehabilitation programmes provide a reasonable alternative to centre‐based cardiac rehabilitation in potentially improving patient participation.^70^ Adjunctive use of digital technologies like personalized smartphone application facilitating patient education, progress tracking and communication between participants and programme staff may also improve adherence to such programmes.^71^, ^72^, ^73^

## The impact of frailty on the efficacy of medical therapy

To date, there has not been a dedicated clinical trial for any drug intervention in highly frail or elderly patients with HF. Most of the data for the efficacy of these interventions in frail patients have been derived from observational studies and post hoc analysis of main trials, which typically enrol younger and healthier patients, more likely to be compliant with therapy (*Table* *2*). A post hoc analysis of the Digitalis Investigation Group (DIG) examined the effects of low (0.5–0.9 ng/mL) and high (≥1 ng/mL) serum digoxin concentration (SDC) in patients aged ≥65 years, versus those aged <65 years in the management of HF.^74^ The analysis found a similar magnitude of benefit in both elderly and younger patients with a significantly higher benefit in patients with low SDC. Hernandez et al. evaluated the efficacy of beta‐blocker initiation following an HF hospitalization in 3001 HFrEF patients and 4153 HFpEF patients aged >65 years over 1 year.^75^ Among the HFrEF cohort with a median age of 80 years, beta‐blocker initiation was significantly associated with a reduction in adjusted all‐cause mortality and all‐cause hospitalizations, while no such benefit was observed in the HFpEF cohort.^75^ Similarly, a propensity‐matched analysis using an HF registry in Japan in elderly patients (median age: 80) revealed that mineralocorticoid receptor antagonist (MRA) use was associated with a significant reduction in the composite endpoint of 1‐year all‐cause mortality and HF hospitalizations, largely attributed to a significant reduction in HF hospitalizations.^76^ A significant benefit was observed in patients with LVEF > 40%, compared to no significant benefit in the LVEF ≤ 40% group. An analysis from the Get With the Guidelines—Heart Failure registry in hospitalized elderly HFrEF patients (>65 years) found that only 10.9% of patients eligible received ARNI after discharge, while 62.0% were prescribed with an ACEi/ARB.^77^ Post‐discharge prescription of ARNI was associated with a reduction in 30‐day and 1‐year mortality and hospitalizations, while a significant difference in outcomes was observed in patients prescribed with an ARNI versus those prescribed with an ACEi/ARB at discharge. A secondary analysis of the DAPA‐HF (Dapagliflozin and Prevention of Adverse Outcomes in Heart Failure) trial evaluated the efficacy of dapagliflozin in HFrEF patients stratified by frailty that was examined as a discrete measure using a 30‐point deficit accumulation index.^78^ A significantly greater reduction in the primary endpoint of HF hospitalizations and cardiovascular death was seen with dapagliflozin in the high frailty group compared to the low frailty group (difference in event rate per 100 person‐years in high frailty −7.9 [95% CI −13.9 to −1.9], intermediate frailty −3.2 [95% CI −6.3 to −0.2] and low frailty −3.5 [95% CI −5.7 to −1.2]). The adverse event rate was high in the high frailty group leading to more frequent discontinuation in both the dapagliflozin and placebo arms.

**Table 2:** Studies evaluating the efficacy of drug therapy in the management of elderly and frail heart failure patients

Study, author	Drug	Study description	Age cut‐off/frailty assessment tool	HFrEF proportion (%)	HFpEF proportion (%)	*N*	Results
Ahmed 2007^74^	Digoxin	A post hoc analysis of the DIG trial in elderly patients aged ≥65 years to evaluate the efficacy of digoxin at high or low serum digoxin levels	Age ≥65	87	13	5548	‐ Reduction in all‐cause mortality, all‐cause hospitalizations and HF hospitalizations in patients with low serum digoxin concentration (0.5–0.9 ng/mL); only reduction in HF hospitalizations with high serum digoxin concentration (≥1 ng/mL)
Hernandez 2009^75^	Beta‐blocker	A retrospective analysis from the OPTIMIZE registry to assess long‐term outcomes in patients aged ≥65 years newly initiated on beta‐blocker therapy	Age ≥65	42	58	24 689	‐ Reduction in all‐cause hospitalizations and mortality compared to those not treated with beta‐blockers
Yaku 2019^76^	MRA	A propensity‐matched analysis from the Kyoto Heart Failure registry evaluating use of MRAs in elderly patients (median age 80) recently hospitalized for HF	Elderly, median age 80	36	64	3717	‐ Reduction in the composite endpoint of HF hospitalizations and mortality in the overall cohort ‐ Subgroup analysis of patients with EF ≤ 40% did not have significant reduction in the primary endpoint
Dewan 2020^5^	ARNI and renin inhibitor	A combined analysis from the PARADIGM and ATMOSPHERE trials in patients stratified by severity to frailty to evaluate the efficacy and safety of ARNI and aliskiren	Rockwood cumulative deficit index	100	0	13 265	‐ No interaction between ARNI and all‐cause death, cardiovascular death or first HF hospitalization across the frailty groups
Greene 2021^77^	ARNI	A retrospective analysis from the Get With the Guidelines—Heart Failure registry evaluating outcomes in hospitalized patients aged ≥65 years with ARNI	Age ≥65	100	0	14 230	‐ Compared to ACEi/ARB, there was a reduced risk of all‐cause mortality in patients receiving ARNI ‐ Compared to patients not on ARNI, there was a reduced risk of all‐cause mortality and hospitalizations in patients receiving ARNI
Butt 2022^78^	SGLT‐2 inhibitor	A post hoc analysis of DAPA‐HF in elderly patients aged ≥65 years with HFrEF to evaluate the efficacy and safety of dapagliflozin	Age ≥65	100	0	4742	‐ Reduction in worsening HF and cardiovascular death across all frailty subgroups stratified by frailty index
Butt 2022^79^	SGLT‐2 inhibitor	A post hoc analysis of DELIVER in patients stratified by severity of frailty to evaluate the efficacy and safety of dapagliflozin	Rockwood cumulative deficit index	0	100	6258	‐ Improvement in KCCQ‐CSS in patients with higher frailty indices ‐ Similar reduction in primary endpoint events across the spectrum of frailty
Butt 2022^15^	ARNI	A post hoc analysis of PARAGON‐HF in patients stratified by severity of frailty to evaluate the efficacy and safety of dapagliflozin	Rockwood cumulative deficit index	0	100	4795	‐ Significant reduction in primary endpoint events of HF hospitalization and cardiovascular death when frailty examined as a continuous measure

Abbreviations: ACEi, angiotensin‐converting enzyme inhibitor; ARB, angiotensin receptor blocker; ARNI, angiotensin receptor–neprilysin inhibitor; DAPA‐HF, Dapagliflozin and Prevention of Adverse Outcomes in Heart Failure; DIG, Digitalis Investigation Group; EF, ejection fraction; HF, heart failure; HFpEF, heart failure with preserved ejection fraction; HFrEF, heart failure with reduced ejection fraction; KCCQ‐CSS, Kansas City Cardiomyopathy Questionnaire—Clinical Summary Score; MRAs, mineralocorticoid receptor antagonists; OPTIMIZE, Organized Program to Initiate Lifesaving Treatment in Hospitalized Patients with Heart Failure; SGLT‐2, sodium–glucose co‐transporter 2.

### Heart failure with preserved ejection fraction

HFpEF is considered a cardio‐geriatric syndrome and has a stronger association with frailty compared to HFrEF. HFpEF is phenotypically characterized by the stiffening of ventricular muscle resulting in diastolic dysfunction with relatively preserved systolic function and is partly considered a sequela of the normal ageing occurring in predominantly older individuals.^80^, ^81^ It is also more prevalent in women, which mirrors the prevalence of frailty stratified by sex in the general HF population.^42^ A post hoc analysis of the TOPCAT (Treatment of Preserved Cardiac Function Heart Failure With an Aldosterone Antagonist) trial evaluated the efficacy of spironolactone in patients with HF and LVEF > 40% stratified by frailty severity as assessed by the deficit accumulation approach.^82^, ^83^ The study found that almost 94% of patients were frail, with a mean FI of 0.37 compared to 0.227 and 0.248 in the PARAGON‐HF and DELIVER (Dapagliflozin Evaluation to Improve the Lives of Patients with Preserved Ejection Fraction Heart Failure) trials.^15^, ^79^ High frailty was associated with significantly higher rates of HF hospitalization and cardiovascular death; however, the benefit of spironolactone on outcomes was not attenuated by frailty severity.

Medical therapy for HFpEF is effective irrespective of the patient's frailty status and may have an amplified benefit in clinical outcomes in frail cohorts. The DELIVER trial evaluated the efficacy of dapagliflozin in patients with HFpEF, HF with mildy reduced ejection fraction (HFmrEF) and those with improved LVEF and found a significant reduction in HF hospitalizations and cardiovascular death with dapagliflozin.^84^ Butt et al., in a secondary analysis of 6258 patients, evaluated if this beneficial effect was attenuated by frailty.^79^ Frailty was assessed using the deficit accumulation approach, and patients were stratified into three FI classes. The analysis showed that higher frailty was associated with a higher rate of primary endpoint with consistent drug efficacy when frailty was measured as a continuous variable (*P* > 0.01). Rates of drug discontinuation and adverse drug reactions were higher in more frail patients. Frail patients also had worse KCCQ‐CSS scores at baseline; however, the improvement in KCCQ‐CSS score after 4 months with dapagliflozin was greater in more frail patients (FI: Class 1, 0.3 [95% CI −0.9 to 1.4]; Class 2, 1.5 [95% CI 0.3–2.7]; and Class 3, 3.4 [95% CI 1.7–5.1] [*P* = 0.021]).

The PARAGON‐HF trial evaluated the efficacy of sacubitril–valsartan in patients with HF and LVEF > 40% and did not find a significant reduction in the primary endpoint of HF hospitalizations and cardiovascular death.^85^ A post hoc analysis was performed in 4795 patients stratified by frailty status using the deficit accumulation approach into three FI classes. Frailty was associated with a higher rate of the primary endpoint, worse QOL scores and increased decline in cognitive function. When examined as a continuous variable, there was a significant interaction between FI and the effect of sacubitril/valsartan, with the most frail receiving the greater benefit (*P* = 0.0032). When evaluated as a discrete variable, the effect was consistent across all three frailty classes (Class 1, rate ratio [RR] 0.98 [95% CI 0.76–1.27]; Class 2, RR 0.92 [95% CI 0.76–1.12]; and Class 3, RR 0.69 [95% CI 0.51–0.95]).

## Initiation, compliance and treatment effects of medical therapy

There is hesitance among physicians to initiate and up‐titrate medical therapy in frail HFrEF patients despite convincing evidence regarding the amplification of benefits in this high‐risk cohort. A retrospective evaluation of 477 ambulatory stable HFrEF patients by Sze et al. revealed that frail patients were less likely to be on foundational HFrEF therapy compared to non‐frail patients (39% vs. 56% of patients were on triple therapy comprising of ACEi/ARB, MRA and beta‐blocker). Furthermore, even if frail patients were on these medications, the doses were significantly suboptimal compared to non‐frail patients.^86^ Worsening frailty over the course of the study period (minimum follow‐up of 1 year) was highly associated with an increased risk of all‐cause mortality and all‐cause hospitalizations, even after adjustment for major confounders including age, body mass index, haemoglobin, NYHA class, Charlson co‐morbidity score and glomerular filtration rate.

A post hoc analysis of the GUIDE‐IT (Guiding Evidence Based Therapy Using Biomarker Intensified Treatment in Heart Failure) trial evaluated the association of frailty with adverse clinical outcomes in patients with HFrEF.^87^ Of the 879 participants in this trial, 56.3% were deemed to have a high FI (>0.310) and were followed up for 12 months for assessment of adverse clinical outcomes.^88^ Patients with high FI were found to have a significantly increased occurrence of the primary composite outcome (43.2% vs. 22.7%; HR 1.76 [95% CI 1.20–2.58]), all‐cause mortality (20.8% vs. 5.5%; HR 2.55 [95% CI 1.25–5.20]) and HF hospitalization (27.6% vs. 21.5%; HR 1.61 [95% CI 1.08–2.40]). The study also assessed the differences in optimization of triple guideline‐directed medical therapy (GDMT; ACEi/ARB, beta‐blocker and MRA) and double GDMT (ACEi/ARB and beta‐blocker) between the non‐frail and highly frail cohorts. Non‐frail were more likely to be started and titrated on both triple GDMT (9.8–28.4%) and double GDMT (38.7–52.6%) compared to patients with high frailty (triple GDMT, 9.3–17.7%; double GDMT, 31.7–40.5%), with similar trends observed with individual components of GDMT (ACEi/ARB and MRA).

Gilstrap et al. primarily evaluated the discontinuation of ARNI compared to ACEi/ARB following hospital discharge and the differential survival benefit over 5 years in patients aged >65 years.^89^ This study modelled a real‐world application on Medicare beneficiaries with HF of drug discontinuation estimates and risk reductions in clinical outcomes using trial‐specific data from the SOLVD (Studies of Left Ventricular Dysfunction), PIONEER‐HF (Comparison of Sacubitril–Valsartan versus Enalapril on Effect on NT‐proBNP in Patients Stabilized from an Acute Heart Failure Episode) and PARADIGM‐HF studies.^44^, ^90^, ^91^ Drug discontinuation rates were found to be higher in patients discharged on ACEi/ARB compared to ARNI (2.3%/month vs. 1.9%/month), with a statistically significant 5‐year survival advantage with ARNI (+0.81 months; 95% CI 0.80–0.81). The effects were consistent with increasing age; the number needed to treat with ARNI compared to ACEi/ARB was 72 overall, 84 in the age group 66–74 and 67 in the age group 85 and above.

Although frail patients are less likely to undergo initiation and up‐titration of medical therapy for HF patients,^87^ GDMT is still safe and effective for these patients.

## Frailty and device therapy

There is a higher prevalence of cardiac resynchronization therapy (CRT) and implantable cardioverter defibrillator (ICD) among older patients^92^; the former is indicated in patients with advanced HF and wide QRS complex to improve interventricular synchrony and reduce morbidity and mortality, whereas the latter is used for primary prevention of sudden cardiac death in patients with persistently low LVEF despite optimal medical therapy.^93^, ^94^ These may be explained by the fact that these patients have had HF for a longer period and tend to have a lower myocardial reserve, lower LVEF and, in case of CRT eligibility, a higher risk of left bundle branch block and atrioventricular dissociation. Data for elderly patients (used as a surrogate indicator of frailty) are mostly derived from post hoc analyses of the main trials, where the median age of all participants is typically <70 years, whereas the real‐world prevalence of CRT devices in patients over the age of 80 years appears to be around 40%.^92^, ^95^ Analyses from the landmark MADIT‐CRT (Multicenter Automatic Defibrillator Implantation Trial with Cardiac Resynchronization Therapy) trial showed that CRT was associated with a significant reduction in the composite endpoint of all‐cause death and HF hospitalizations among participants within age groups 60 to 74 and 75 and above, while no significant difference was observed in patients aged 60 years and lower.^96^, ^97^ There is also a scarcity of prospective studies that evaluate the efficacy and safety of therapeutic devices in patient populations stratified by objective frailty assessment methods, rather than using age as a surrogate marker, as performed for several therapeutic drugs discussed earlier. Device safety is also questionable in frail patients as they may be more prone to peri‐procedural and post‐procedural adverse events, which are not commonly observed in younger, less frail, low‐risk patients. For example, 10–20% of patients with an ICD in place experience inappropriate shocks, which have lasting effects on patients' physical and mental health, which may be more pronounced in older, more frail patients.^98^ Similarly, lead infections associated with CRT and ICD have a high morbidity and mortality rate, requiring prolonged hospitalizations and multiple procedures for extraction and replacement of infected lead/device. More invasive procedures like left ventricular assist device placement and heart transplantation in patients with frailty are associated with lower survival and increased length of intensive care unit and hospital stay.^99^, ^100^ Therefore, a careful assessment of benefits and risk profiles is warranted in eligible patients for various device therapies, as both benefits and risks may be amplified in this high‐risk cohort.

## Challenges associated with the management of heart failure in frail patients

The association between HF and frailty is cyclical; HF imposes significant physical limitations through decreased exercise tolerance and physical deconditioning, which contribute to increased frailty. On the other hand, existing frailty as an independent variable further worsens physical limitations in HF and may also increase the risk of incident HF. The frail HF population represents a high‐risk cohort that may benefit from medical and device therapies but is commonly not deemed to be eligible due to a perceived notion of therapeutic futility and increased risk of harm associated with any disease‐modifying intervention. A high co‐morbidity burden, for example, chronic kidney disease, osteoarthritis, cancer and dementia, more commonly seen in frail and the elderly is a significant hurdle to starting and optimizing medical therapy and can complicate initiation and up‐titration of medical therapies, which have multi‐system adverse events. However, clinical inertia further propagates this notion of exaggerated perceived harm in most cases leading to potential undertreatment of this vulnerable population.

Current evidence suggests that most therapies have consistent benefits in non‐frail and frail patients. These findings need further reinforcement in guideline recommendations, so there is little hesitancy to initiate or continue disease‐modifying interventions in this high‐risk population. There is a need for development of HF‐specific frailty assessment tools to assess patients at risk for or severity of existing frailty and inculcation of these tools in routine clinical practice to promptly identify meaningful changes in frailty and health status to guide management decisions. HF hospitalization is a critical period in the disease trajectory with frail patients being at highest risk for continued deterioration^101^; hence, it is pivotal to implement treatment strategies for HF including cardiac rehabilitation and aggressively target contributory chronic co‐morbidities in both stable, ambulatory patients and those with worsening HF to ensure preservation of health status and attenuation of adverse outcomes. Moreover, because there is still limited evidence for treatments for older, frail HF patients, a concerted effort is required to perform dedicated trials in these patients for evaluation of treatment strategies as the magnitude of treatment effect in this cohort may be higher compared to younger, non‐frail patients. Reduction in symptom burden and hospitalization and improved physical/functional capacity by institution of appropriate therapies in HF can lead to an improvement in frailty status as both clinical syndromes are interlinked as described earlier. However, frailty should also be treated as its own separate multi‐domain entity inclusive of physical, cognitive and social frailty. It is also important to emphasize that frailty is potentially reversible or can be delayed with timely interventions.^102^

## Conclusions

While frailty is an important patient‐level factor that negatively affects prognosis in HF and coexists with and complicates other clinical co‐morbidities, it is also considered a major barrier to the initiation of life‐saving HF therapies. Frail patients are considered at higher risk for adverse events from the comprehensive implementation of medical therapy and appropriate institution of device therapy; however, paradoxically, it appears that these are patients who would benefit most from these interventions. The current body of evidence suggests that it may be relatively safe and efficacious to initiate most treatment strategies in frail patients with HF, and the multi‐modal therapeutic approach to HF needs to be individualized based on the severity and nature of frailty. However, the evidence needs to be further consolidated with dedicated trials recruiting older, more frail patients for these specific therapies to assess comparative effectiveness in pre‐specified health‐related QOL endpoints, mortality and hospitalizations, and safety in this high‐risk cohort.

## Conflict of interest statement

Dr. Pandey is supported by the Texas Health Resources Clinical Scholarship, the Gilead Sciences Research Scholars Program, the National Institute on Aging GEMSSTAR Grant (1R03AG067960‐01) and Applied Therapeutics; has served on the advisory board for Roche Diagnostics; and has received nonfinancial support from Pfizer and Merck. Dr. Fudim was supported by the American Heart Association (20IPA35310955), Doris Duke, Bayer, Bodyport and Verily. He receives consulting fees from Abbott, Ajax, Alio Health, Alleviant, Audicor, Axon Therapies, Bayer, BodyGuide, Bodyport, Boston Scientific, Broadview, Cadence, Cardionomics, Coridea, CVRx, Daxor, Deerfield Catalyst, Edwards Lifesciences, EKO, Feldschuh Foundation, FIRE1, Gradient, Hatteras, Impulse Dynamics, InterShunt, Medtronic, NI Medical, NXT Biomedical, Pharmacosmos, PreHealth, ReCor, Shifamed, Splendo, Summacor, SyMap, Verily, Vironix, VisCardia and Zoll outside the submitted work. Dr. Butler reported personal fees from Abbott, Adrenomed, American Regent, Amgen, Applied Therapeutics, Array, AstraZeneca, Bayer, Boehringer Ingelheim, Cardior, CVRx, Eli Lilly and Company, G3 Pharma, Imbria, Impulse Dynamics, Innolife, Janssen, LivaNova, Medtronic, Merck, Novartis, Novo Nordisk, Occlutech, Pfizer, Relypsa, Roche, Sanofi, Sequana Medical and Vifor for consulting outside the submitted work. Dr. Anker reported grants from Abbott Vascular and Vifor International and personal fees from Abbott Vascular, Amgen, AstraZeneca, Bayer, Boehringer Ingelheim, BioVentrix, Brahms, Cardiac Dimensions, Cardior, Cordio, CVRx, Edwards Lifesciences, Impulse Dynamics, Janssen, Novartis, Occlutech, Respicardia, Servier, Thermo Fisher Scientific, Vectorious, Vifor and V‐Wave outside the submitted work. Dr. Talha and Dr. Khan have no relevant disclosure.
